# Association between variation in the genes DDAH1 and DDAH2 and hypertension among Uygur, Kazakh and Han ethnic groups in China: Associação entre variação de genes DDAH1 e DDAH2 com hipertensão nos grupos étnicos uygur, kazakh e han na China

**DOI:** 10.1590/1516-3180.2015.01150108

**Published:** 2016-01-19

**Authors:** Zhong Wang, Shaoze Chen, Lina Zhang, Guilin Lu, Chengming Zhou, Dao Wen Wang, Li Wang, Bayinbate Badengmu, Zhihong Zhai, Lian Qin

**Affiliations:** I MD. Head of Department, Department of Cardiology, First Affiliated Hospital, Shihezi Medical College, Shihezi University, Shihezi, China.; II MD. Head of Department, Department of Cardiology, First Affiliated Hospital, Shihezi Medical College, Shihezi University, Shihezi, China. Doctoral Student, Department of Internal Medicine and Genetic Diagnosis Center, Tongji Hospital, Tongji Medical College, Huazhong University of Science and Technology, Wuhan, China.; III MD. Doctoral Student, Department of Internal Medicine and Genetic Diagnosis Center, Tongji Hospital, Tongji Medical College, Huazhong University of Science and Technology, Wuhan, China.; IV MD. Head of Department, Department of Cardiology, The People ‘s Hospital of Boertala, Bole, China.

**Keywords:** Cardiovascular system, Blood pressure, Hypertension, Genes, Polymorphism, Genetic

## Abstract

**CONTEXT AND OBJECTIVE:**

Dimethylarginine dimethylaminohydrolase enzymes (DDAH), which are encoded by the genes DDAH1 and DDAH2, play a fundamental role in maintaining endothelial function. We conducted a case-control study on a Chinese population that included three ethnic groups (Han, Kazakh and Uygur), to systemically investigate associations between variations in the genes DDAH1 and DDAH2 and hypertension.

**DESIGN AND SETTING:**

Experimental study at the Department of Internal Medicine and Genetic Diagnosis, Tongji Hospital, Tongji Medical College, Huazhong University of Science and Technology.

**METHODS:**

This case-control study included 1,224 patients with hypertension and 967 healthy unrelated individuals as controls. DDAH1 -396 4N (GCGT) del>ins, rs3087894, rs805304 and rs9267551 were genotyped using the TaqMan 5’ nuclease assay.

**RESULTS:**

The G/C genotype of rs3087894 in DDAH1 was a risk factor for hypertension in the Kazakh group in the co-dominant model (G/C versus G/G) (OR 1.39; 95% CI: 1.02-1.88; P < 0.05), with the same result in the dominant model (G/C + C/C versus G/G) (OR 1.38; 95% CI: 1.03-1.84; P < 0.05). In contrast, the C/C genotype of rs3087894 seemed to be a protective factor against hypertension in the Uygur group in the recessive model (C/C versus G/G + G/C) (OR 0.62; 95% CI: 0.39- 0.97; P < 0.05). Similar findings for rs3087894 were also observed after adjusting the variable for the age covariate.

**CONCLUSION:**

Our results indicated that the C-allele of rs3087894 in DDAH1 was a risk factor for hypertension in the Kazakh group but a protective factor in the Uygur group.

## RESUMO

**CONTEXTO E OBJETIVO:**

Enzimas dimetilarginina dimetilaminohidrolase (DDAH), codificadas por genes DDAH1 e DDAH2, desempenham papel fundamental na manutenção da função endotelial. Realizamos estudo tipo caso-controle na população chinesa, com três grupos étnicos (han, kazakh e uygur) para investigar sistematicamente a associação entre a variação de genes DDAH1 e DDAH2 e a hipertensão.

**DESENHO E LOCAL:**

Estudo tipo caso-controle no Departamento de Medicina Interna e Diagnóstico Genético, Hospital de Tongji, Tongji Medical College, Universidade de Ciência e Tecnologia de Huazhong.

**MÉTODOS:**

Este estudo incluiu 1.224 pacientes com hipertensão e 967 indivíduos saudáveis, sem parentesco, como controles. DDAH1 -396 4 N (GCGT) del > ins, rs3087894, rs805304 and rs9267551 foram genotipados usando o ensaio nuclease TaqMan 5’.

**RESULTADOS:**

O genótipo G/C de rs3087894 no DDAH1 foi um fator de risco para a hipertensão arterial no grupo kazakh em modelo codominante (G/C *versus* G/G; OR 1,39; IC 95%: 1,02-1,88; P < 0,05), com o mesmo resultado no modelo dominante (G/C + C/C *versus* G/G; OR 1,38; IC 95%: 1,03-1,84; P < 0,05). Em contraste, o genótipo C/C de rs3087894 parecia ser um fator de proteção para a hipertensão no grupo uygur no modelo recessivo (C/C *versus* G/G + G/C; OR 0,62; IC 95%: 0,39-0,97; P < 0,05). Achado semelhante para rs3087894 também foi observado depois de se ajustar a variante à covariante idade.

**CONCLUSÃO:**

Os nossos resultados indicaram que o C-alelo de rs3087894 no DDAH1 foi fator de risco para a hipertensão no grupo de kazakh, mas fator de proteção no grupo de uygur.

## INTRODUCTION

Raised blood pressure (BP) or hypertension is a multifactorial disorder and a major risk factor for stroke and ischemic heart disease. The World Health Organization has estimated that hypertension affects over one billion people and that will rise to 1.5 billion by 2020, thus contributing 13.5 million deaths worldwide annually.^1^^,^^2^^,^^3^ Hypertension is a complex trait influenced by genetic or environmental factors (including age, weight, ethnicity and diet), or their interactions.^4^^,^^5^^,^^6^^,^^7^^,^^8^ Many studies have demonstrated that estimates of heritability of BP range from 31% to 68%,^9^^,^^10^^,^^11^ and this has prompted extensive efforts to identify genes associated with hypertension. A legible genetic map of hypertension will highlight potential drug targets for its prevention or treatment and reduce the risk of cardiovascular events among people with hypertension.^12^^,^^13^ A large number of candidate genes for hypertension have now been widely studied and, among these, the genes DDAH1 and DDAH2 seem to have an influence on hypertension, since they play a fundamental role in maintaining endothelial function.^14^^,^^15^^,^^16^

Dimethylarginine dimethylaminohydrolase enzymes (DDAH), which are encoded by the genes DDAH1 and DDAH2, clear asymmetric dimethylarginine (ADMA) from the kidneys and liver.^17^ ADMA, as an endogenous inhibitor of nitric oxide (NO) synthase, reduces NO and thus regulates endothelial function. Based on detection of endothelial dysfunction at the origin of hypertension, ADMA has considerable clinical impact on hypertension. Studies have demonstrated that DDAH1 variants are associated with increased plasma ADMA and increased risk of thrombosis, stroke and coronary artery disease (CAD) in humans.^18^^,^^19^ Similarly, Leiper et al. have shown that loss of DDAH1 activity leads to accumulation of ADMA and reduction in NO signaling, which in turn causes vascular pathophysiology, including endothelial dysfunction, increased systemic vascular resistance and elevated systemic and pulmonary blood pressure.^20^

## OBJECTIVE

In view of the genetic heterogeneity among ethnic groups, in the present study we aimed to systemically evaluate associations between the DDAH1 and DDAH2 variants and hypertension in three ethnic groups within the Chinese population (Han, Kazakh and Uygur).

## METHODS

### Subjects

This case-control study included 1,224 patients with hypertension (660 males and 564 females; mean age 55.90 ± 11.23 years) who had histories of hypertension (systolic BP ≥ 140 mmHg and/or diastolic BP ≥ 90 mmHg) or histories of use of anti-hypertension medication such as calcium channel blockers, beta-blockers, angiotensin receptor blockers, angiotensin-converting enzyme inhibitors or diuretics. Patients with kidney disease (glomerular filtration rate, GFR, of less than 60 ml/minute) were excluded from the study. In addition, a group of 967 healthy unrelated individuals (504 males and 463 females; mean age 51.62 ± 13.50) without hypertension was studied as a control group. The control group was initially recruited in the same study and was matched to cases according to race, geographic location and date of data-gathering (within three months). Individuals from three ethnic groups (Han, Kazakh and Uygur) who came to Shihezi hospital or outpatient clinic, in Xinjiang province, China, between January 2013 and December 2013, were recruited. All participants signed an informed consent statement. This study was approved by the Medical Ethics Committee of the hospital and the study protocol conformed to the ethical guidelines of the 1975 Declaration of Helsinki.

All participants underwent standard medical history-taking and physical evaluations. They answered a questionnaire that sought complete demographic information (name, age, sex, smoking, drinking, history of disease and so on). A complete general physical examination was carried out. BP was measured in a sitting position using a standard analogue sphygmomanometer. Plasma glucose, lipid and homocysteine levels were measured using standard methods, using an Olympus AU 2700 automatic biochemical analyzer (Olympus CO Ltd., Tokyo, Japan), after the patients had fasted for 12 h overnight.

### DNA extraction and genotyping

Genomic DNA from whole blood containing EDTA was isolated using a DNA isolation kit (Tiangen Biotech, Beijing, China), in accordance with the protocol. DDAH1 -396 4N (GCGT) del>ins, c.17 G > C (rs3087894), DDAH2 c.531 A > C (rs805304) and c.662 G > C (rs9267551) were genotyped using the TaqMan 5’ nuclease assay in the 7900HT fast real-time PCR system, using primers and probes synthesized by ABI (Applied Biosystems, Foster City, CA, USA), under standard conditions.^21^ The sequences of probes and primers for DDAH1 -396 4N (GCGT) del>ins were as follows: forward primer, 5’ CAGGTAAAGACCAGGAAGCCC 3’; reverse primer, 5’ GGACCTCGGCGAAAAGC 3’; probe 1 del, 5’ CGCAGGTGCACAC 3’; and probe 2 ins, 5’ AGGTGCACGCACAC 3’. The product codes of the other TaqMan probes were, in turn, C_2518300_10, C_3233671_10 and C_27848488_10.

### Statistical analysis

Continuous variables were shown as mean values, with their standard deviation and categorical variables expressed as absolute numbers with their prevalence. The Kolmogorov-Smirnov test was used to evaluate departure from normal distribution, and this was evaluated for all continuous variables. The independent-sample t test and Pearson’s chi-square test were applied in order to observe the differences between patient and control groups for continuous variables and categorical variables, respectively. The data relating to genotype frequencies of DDAH1 and DDAH2 variants in the patients and controls were obtained by means of direct counting, and significant differences among ethnic subgroups were assessed using Pearson’s chi-square test or Fisher’s exact test. The Hardy-Weinberg equilibrium was evaluated using Pearson’s chi-square test in order to determine the variation in distribution of alleles and genotypes within the study population. Inheritance hypotheses were tested in accordance with three models: co-dominant, dominant and recessive. Binary logistic regression was built to determine associations between the DDAH1 and DDAH2 variants and hypertension.

All statistical analyses were performed using the Statistical Package for the Social Sciences (SPSS Inc., Chicago, IL, USA), version 17.0. Double-sided P-values < 0.05 were considered statistically significant.

## RESULTS

### Characteristics of the study population

This case-control study recruited 1,224 patients with hypertension and 967 controls. Clinical information was collected at baseline from all subjects and was compared between patients and controls. The clinical characteristics and laboratory test data are shown in Table 1. No differences relating to sex, ethnicity, smoking or drinking were found between the patient and control groups (P > 0.05). The mean age of the patients was significantly higher than that of the controls (55.90 ± 11.23 versus 51.62 ± 13.50; P < 0.01). The histories of CAD, stroke and diabetes differed significantly between patients and controls (P < 0.05). In contrast with the controls, systolic BP, diastolic BP and body mass index were clearly higher among the patients. Moreover, the glucose, triglyceride, total cholesterol, low-density lipoprotein cholesterol, apolipoprotein B and homocysteine levels were significantly higher among the patients (P < 0.05). There were no differences between the two groups regarding apolipoprotein A1 and high-density lipoprotein cholesterol.

**Table 1.**
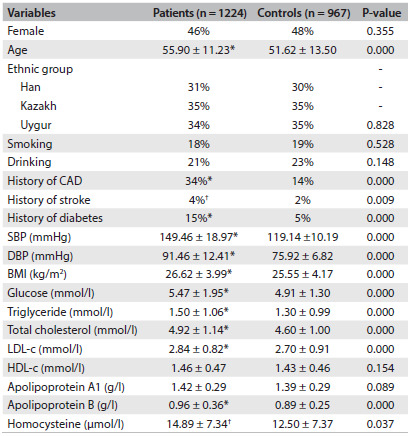
Baseline characteristics of patients and controls

*indicates P-value < 0.01; ^†^indicates P-value < 0.05; CAD = coronary artery disease; SBP = systolic blood pressure; DBP = diastolic blood pressure; BMI = body mass index; LDL-c = low-density lipoprotein cholesterol; HDL-c = high-density lipoprotein cholesterol.

### Genotype distribution in the three ethnic groups

Before analyzing gene variation in relation to hypertension, it was noteworthy that the genetic background was markedly different between the ethnic groups. Based on the TaqMan results, we determined and recorded the genotype of each individual and calculated the genotype and allele frequencies of each variation (DDAH1 -396 4N de l > ins, c.17 G > C, DDAH2 c.531 A > C and c.662 G > C) manually for the Han, Kazakh and Uygur groups (Table 2). The genotype distribution of the four variations was in line with the Hardy-Weinberg equilibrium (P > 0.05). The genotype distribution of all four variations except for DDAH2 c.531 A > C was significantly different in the three ethnic groups and the minor allele frequency (MAF) was lower in the Han group than in the Kazakh and Uygur groups.

**Table 2.**
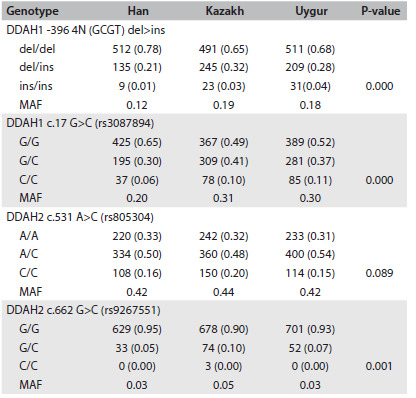
Genotype distribution of DDAH1 and DDAH2 polymorphisms in Han, Kazakh and Uygur groups

Data are shown as genotype number (proportion of genotype). P-values were obtained from comparisons between the three ethnic groups using Pearson’s chi-square test. MAF = minor allele frequency.

### Association between gene variation and hypertension

To evaluate associations between gene variation (DDAH1 -396 4N del > ins, c.17 G > C, DDAH2 c.531 A > C and c.662 G > C) and hypertension, we performed logistic regression analysis in accordance with three inheritance models: co-dominant, dominant and recessive. The results showed that there was no significant difference between hypertension patients and controls, irrespective of the ethnic factor.

Next, we assessed the effect of gene variation on hypertension in an ethnicity-specific case-control analysis. The G/C genotype of rs3087894 (c. 17 G > C) in DDAH1 was a risk factor for hypertension in the Kazakh group in the co-dominant model (G/C versus G/G) (OR = 1.39; 95% CI = 1.02-1.88; P < 0.05) and the same result was obtained in the dominant model (G/C + C/C versus G/G) (OR = 1.38; 95% CI = 1.03-1.84; P < 0.05). In contrast, the C/C genotype of rs3087894 seemed to be a protective factor against hypertension in the Uygur group in the recessive model (C/C vs. G/G + G/C) (OR = 0.62; 95% CI = 0.39-0.97; P < 0.05), whereas there were no differences between patients and controls in the Han group (P > 0.05). No significant difference was observed in relating to the other three gene variation. The logistic regression and OR results, for possible associations between hypertension and gene variation are shown in Table 3. Similar findings for rs3087894 were also observed after adjusting the variable for the age covariate (Table 4).

**Table 3.**
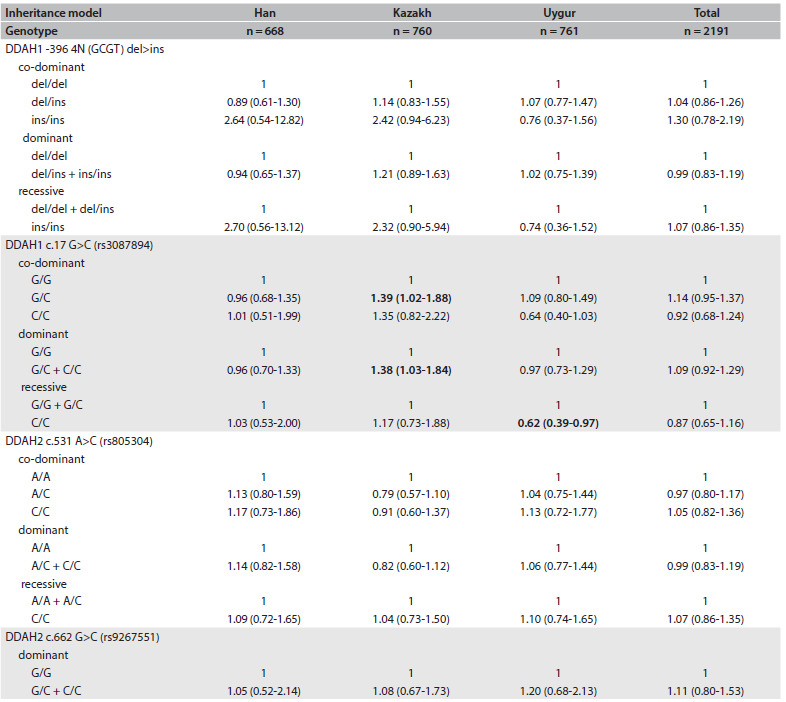
Odds ratios of DDAH variation in relation to hypertension in the Han, Kazakh and Uygur groups

Data are shown as odds ratio (with 95% confidence intervals). Data in bold and italic are binary logistic results between case-control groups that showed statistically significant differences (P < 0.05).

**Table 4.**
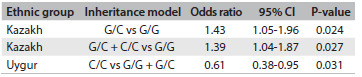
Association between rs3087894 in DDAH1 and hypertension in the Kazakh and Uygur groups after adjusting for the age covariate

CI = confidence interval

## DISCUSSION

To our knowledge, this was the first investigation of an association between variation in the genes DDAH1 and DDAH2 and hypertension in a multi-ethnic Chinese population. The main finding from this study was that it demonstrated that the C-allele of rs3087894 in DDAH1 is a risk factor for hypertension in the Kazakh group but a protective factor in the Uygur group. In addition, we did not find any genotype of DDAH1 and DDAH2 associated with hypertension in the Han group.

DDAH may play a crucial role in blood pressure regulation through clearance of ADMA, which inhibits NO synthase, thus having implications with regard to development of atherosclerosis.^22^ Some studies, both on animals and on humans, have demonstrated that DDAH activity has a critical role in regulating NO synthesis *in vivo*. Hu et al. found that plasma and tissue ADMA levels in DDAH1 knockout mice were several times higher than in wild-type mice.^23^ In human research, previous studies have shown that more than 70% of the clearance of ADMA was due to DDAH activity and that genetic variation in DDAH1 was related to ADMA levels.^24^^,^^25^ These studies have shown that there could be functional variants in DDAH genes and that this is likely to affect the risk of vascular events.^18^ Ding et al. found that DDAH1 polymorphism (-396 4N del>ins) in the promoter region was associated with increased risk of thrombosis, stroke and coronary heart disease (CHD) in Han groups in China.^19^ Maas et al. found that the -1151 A/C and -449 G/C polymorphisms in the DDAH2 promoter region were associated with increased prevalence of hypertension.^26^

In the present study, we hypothesized that genetic variation in DDAH1 (-396 4N del > ins and c.17 G > C) and DDAH2 (c.531 A > C and c.662 G > C) would present an association with hypertension, and we tested this in three ethnic groups. Because of the different genetic backgrounds, the genotype distribution of all four variations except for DDAH2 c.531 A > C was significantly different between the three ethnic groups, and MAF was lower in the Han group than in the Kazakh and Uygur groups.

Our study highlighted conflicting results for rs3087894 in DDAH1 in the three ethnic groups. We found that the C-allele of rs3087894 was a risk factor for hypertension in the Kazakh group (G/C versus G/G: OR = 1.39; 95% CI = 1.02-1.88; G/C + C/C versus G/G: OR = 1.38; 95% CI = 1.03-1.84; P < 0.05) but a protective factor in the Uygur group (C/C versus G/G + G/C: OR = 0.62; 95% CI = 0.39 - 0.97; P < 0.05). This was not a significant factor in the Han group (P > 0.05).

The Uygur, Kazakh and Han groups are the three main ethnic groups living in the Xinjiang area, and they have total different genetic background, lifestyle and eating habits. Han individuals in this area have usually migrated from other provinces of China and have the typical way of life of the Han people. The Uygurs and Kazakhs are natives of Xinjiang. Uygurs are mainly engaged in farming and trade, but most Kazakhs still live a nomadic life. Moreover, Kazakhs have the highest incidence of hypertension among these three ethnic groups, while Uygurs are the contrary.

In our study, we found that the C-allele of rs3087894 in DDAH1 plays conflicting roles in these three ethnic groups. The mechanisms of this association need further investigation. The most possible explanation for the discrepancies lies within differences in linkage-disequilibrium structure between these ethnic backgrounds. Differences in gene-gene or gene-environment interactions in relation to the development of hypertension in these ethnic groups may also contribute towards the discrepancies. Lastly, the small sample size may also had had a certain degree of influence on the research results. Further research, specifically large-scale prospective cohort studies and further gene linkage-disequilibrium investigations, will be needed in order to confirm these findings.

## CONCLUSION

We identified that rs3087894 in DDAH1 was significantly associated with hypertension and showed conflicting results in different ethnic groups. This is therefore a candidate for further studies with the aim of helping to ascertain the mechanisms of hypertension in different populations.
